# Discovering the mesoscale for chains of conflict

**DOI:** 10.1093/pnasnexus/pgad228

**Published:** 2023-08-01

**Authors:** Niraj Kushwaha, Edward D Lee

**Affiliations:** Complexity Science Hub, Josefstædter Strasse 39, 1080 Vienna, Austria

**Keywords:** armed conflict, transfer entropy, causal network, scaling

## Abstract

Conflicts, like many social processes, are related events that span multiple scales in time, from the instantaneous to multi-year development, and in space, from one neighborhood to continents. Yet, there is little systematic work on connecting the multiple scales, formal treatment of causality between events, and measures of uncertainty for how events are related to one another. We develop a method for extracting causally related chains of events that addresses these limitations with armed conflict. Our method explicitly accounts for an adjustable spatial and temporal scale of interaction for clustering individual events from a detailed data set, the Armed Conflict Event & Location Data Project. With it, we discover a mesoscale ranging from a week to a few months and tens to hundreds of kilometers, where long-range correlations and nontrivial dynamics relating conflict events emerge. Importantly, clusters in the mesoscale, while extracted from conflict statistics, are identifiable with mechanism cited in field studies. We leverage our technique to identify zones of causal interaction around conflict hotspots that naturally incorporate uncertainties. Thus, we show how a systematic, data-driven, and scalable procedure extracts social objects for study, providing a scope for scrutinizing and predicting conflict and other processes.

**Significance Statement**

While infamous wars like World War I are seemingly coherent, they are actually composed of a variety of events that have been clustered together by choice. How they should cluster is complicated because events may be instantaneous like an explosion but may result from years of building tension. We address this problem by developing a statistical approach for uncovering chains of related conflict events that accounts for scale. We discover that coherent chains of conflict events predominate in a specific mesoscale. Within this mesoscale, our approach connects events in a way that highlights plausible and potentially hidden mechanism. Our method helps uncover and exclude causal links in conflict spread and can be applied to other spreading social phenomena.

Historically, the study of armed conflict has focused on predefined aggregates like skirmishes, battles, and wars, where individual acts of violence have been integrated into a coherent whole by experts (1–3). Yet, such a procedure is difficult to replicate systematically across time periods and regions because it is fundamentally qualitative. More recently, sensitivity to the underlying assumptions in the definition of conflict has inspired the creation of “disaggregated” data sets, where the atomic units, or events, are delimited by a location, time, and other distinguishing characteristics (4, 5). Naturally, disaggregated data introduce the complementary difficulty of clustering events into meaningful conflict aggregates (5, 6). Generally, heuristics are used to group events together using properties like involved actors (7), geographical boundaries (8), administrative boundaries (9), or ethnic divisions (10). While these approaches are helpful for building intuition, they are not considered systematic in the conflict literature (4, 8, 11, 12), the groupings are fixed and rigid, and they can be sensitive to the way that the data are labeled, which is subject to purposeful or inadvertent errors. In short, there is a need for a systematic procedure for dealing with scale that goes beyond qualitative treatments (12), a quantitative framework for extracting causal relationships, and consequently a provision for uncertainty in the inferred relationships between conflict events. Such a technique would be useful not only for the study of political violence but more generally for other social processes that spread across time and space.

We demonstrate here a systematic procedure that addresses these limitations by uncovering causal patterns from conflict statistics. Our approach is inspired by fundamental advances in physics and biophysics relating to the analysis of multiple scales in cascades such as the propagation of stress in collapsing materials and neural activity in the brain (13–17). Our approach is robust to errors because it relies only on information about the presence or absence of conflict, introduces a distance-dependent measure of causal interaction incorporating uncertainties, and allows analyses to move systematically between spatial and temporal scales.

We focus on the Armed Conflict Location & Event Data Project (ACLED), which provides an extensive, publicly available, and disaggregated dataset on worldwide conflict (4). Each conflict event noted in the database occurs in a particular time and place between a set of actors constituting a point of activity as plotted on the map in Fig. 1. By summing over the points of activity in a particular region, we are also able to track levels of conflict over time as in the insets. Information about conflict events is collected from news and local sources, and the database details for each event alleged actors, fatalities, location, date, and precision of the provided data. Importantly, the events between armed groups are labeled as “battles,” allowing us to focus on them. Amongst the battles, we analyze conflict in Africa because it is there where we have the longest observational period (1997–2019) and a large contiguous landmass compared to other regions. As a result, the data set provides a high-resolution perspective on the atomic units of conflict that we can use to determine how events should be joined together.

**Fig. 1.**
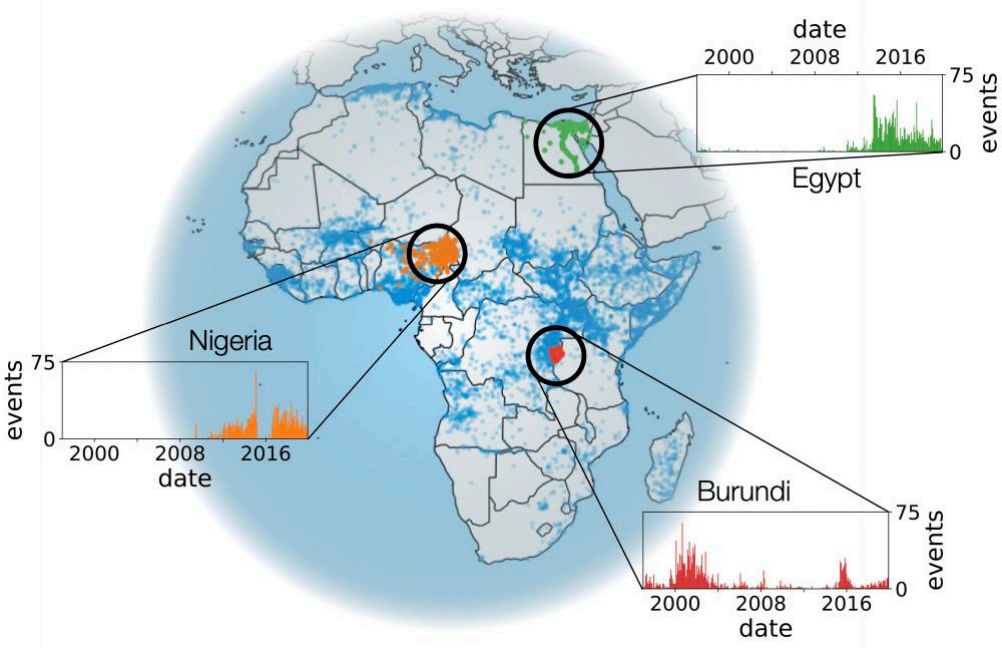
Spatial and temporal distribution of conflict events included in the Armed Conflict Location & Event Data Project (ACLED) from 1997 through 2019 in Africa. Each point is a location at which conflict has been reported. For the three regions of northeastern Nigeria, Egypt, and Burundi, we show the monthly incidence of reported conflict events.

As an example of what one would like to analyze, we highlight attacks labeled “Boko Haram” in northeastern Nigeria in orange in Fig. 1. Neither grouping conflicts by major militant group Boko Haram nor by country boundaries captures their relationship to surrounding areas; for example, field research has indicated that the group drives conflict in western Chad by forcing herders to migrate south and east (18). At a wider scale, violence perpetrated by Boko Haram impacts conflict prevalence elsewhere in Nigeria, if indirectly, because such events tend to sap government resources, erode government legitimacy, and cause economic damage (19–21), connections that are not apparent from this grouping. Inspecting the spatial distributions in Fig. 1, we see that conflict events tend to cluster with one another in time and space (22). This suggests that the way that conflict may drive more conflict would be detectable in local statistical patterns of activity.

We leverage local conflict patterns to extract a causal geographic web identifying paths through which conflicts might affect each other. Building on previous work, we set spatial and temporal separation scales, *b* and *a*, grouping together conflict events that fall within the specified distance of one another (22). This is akin to establishing a minimal resolution in our viewing lens, or a scale on a spatial kernel or temporal memory, such that events that are closer together than this distance cannot be distinguished from one another. Here, we perform such a discretization using temporal bins of duration *a* and pseudorandom Voronoi cells with typical radius *b* to avoid artifacts from regular lattices. We show examples of the cells in Fig. 2 (more details on the algorithm in Appendix B). Our procedure allows us to titrate the coarseness of our resolution with precisely defined scales, at the smallest scales grouping only local conflict events together and at the largest allowing for the possibility that conflict events belong together across continental distances and years.

**Fig. 2.**
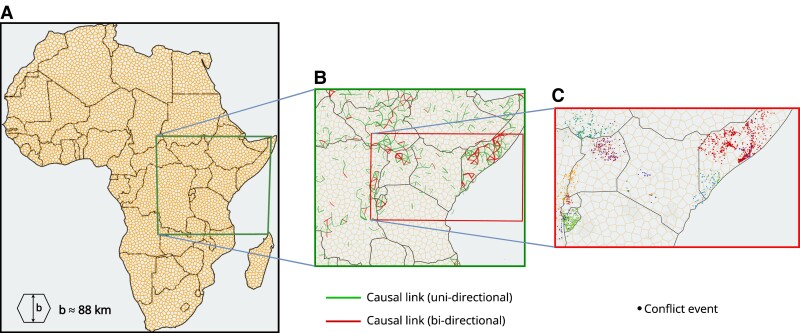
Illustration of conflict avalanche generation (see Appendix B for detailed algorithm). A) We first set the spatial and temporal separation scales. B) We then infer causal structure by calculating directed transfer entropy for pairs of neighboring spatial bins. An example of the causal network is shown for temporal scale a=64 days and spatial scale b≈88 km. Links are directed in nature but the arrows are not shown here for simplicity. C) Conflict avalanches are sequences of conflict events (points on map) that are connected through the causal network. Different colors correspond to different avalanches.

For a given scale, we determine whether or not a particular Voronoi cell $x_{t}$ had any conflict at some moment in time bin indexed *t* in which case $x_{t}=1$; otherwise, $x_{t}=0$. This presents a binary time series, where the pattern of activity reveals interaction between conflict in time and space. As a pragmatic hypothesis that limits the range of possible causal interactions (and thus many false positives and negatives), we start with the assumption of local causality, or that conflicts in one zone *x* are potentially affected only by neighboring zones *y* defined as cells that touch. The simplest case is if no neighboring influence exists such that conflict at one site influences itself in the future (self-influencing sites in Africa shown in Fig. S3). In other words, we would expect that if the presence of conflict in the future of site *x*, denoted as $x_{t+1}$, depended on the past $x_{t}$, then the relationship between the joint probabilities $q(x_{t},x_{t+1})$ would not factorize into the marginals, or $q(x_{t},x_{t+1})\neq q(x_{t})q(x_{t+1})$. This difference is given by the mutual information between past and future (23)


(1) I[Xt;Xt+1]=∑xt∈{0,1}xt+1∈{0,1}q(xt,xt+1)log(q(xt,xt+1)q(xt)q(xt+1)).

On the other hand, it could have been the case that the neighborhood played a role such that having information about a neighbor at the present $y_{t}$ helps predict what happens in the future $x_{t+1}$. This is exactly the quantity described by the transfer entropy (24), which tells us if knowing about neighboring cell $y_{t}$ conveys any further information about $x_{t+1}$ beyond what was already given by $x_{t}$,


(2) $$T[X;Y]=\sum_{x_{t},x_{t+1},y_{t}}q(x_{t},x_{t+1},y_{t})\log\;\left(\frac{q(x_{t+1}|x_{t},y_{t})}{q(x_{t+1}|x_{t})}\right).$$

Transfer entropy is zero when $q(x_{t+1}|x_{t},y_{t})=q(x_{t+1}|x_{t})$. Unlike Granger causality, transfer entropy is a nonlinear and general measure of statistical dependence and the two are equivalent only for Gaussian variables (25). Finally, we must worry about the fact that we have a finite time series on which to calculate Eqs. 1 and 2. To take this into account, we test the significance of the mutual information and transfer entropy measures by asserting that they are only significantly different from zero when at least a fraction 1−p of time-shuffled values are smaller than the measured value (26) (see Fig. S5 for the distributions of transfer entropy and mutual information). As we adjust the significance threshold *p*, we go from allowing any pair of proximate conflict events to be connected, p=1, to no connections, p=0. Here, we only take edges as potential candidates of causal connection when they are significant with the cutoff p≤1/20. This dramatically limits the number of candidates—ones that we later show are plausibly tracking causal mechanism—indicating that only a sparse set of the possible edges between cells display signal.

The candidate causal connections that we find tend to cluster in geographic regions including the Sahel from multiple ongoing conflicts (27, 28), northeastern Nigeria from Boko Haram (29), Nigeria and Cameroon from Ambazonian Separatist (30), Northern Africa from civil war (31), the Horn of Africa from state failure and insecurity (32), the Darfur region from genocide and ethnic hostilities (33), Angola and Congo from civil war (34), and Madagascar from the Dahalo Militia (35) as we show in Fig. 3a. In contrast, a null model where we have time shuffled all the events in each Voronoi cell leads to a dispersed and fragmented causal network as we show in Fig. 3B (see Fig. S4 for space shuffled null model). That the highly connected regions represent recognizable conflict “hotspots” confirms the power of our systematic procedure only accounting for statistical signatures of causality in observed conflict events.

**Fig. 3.**
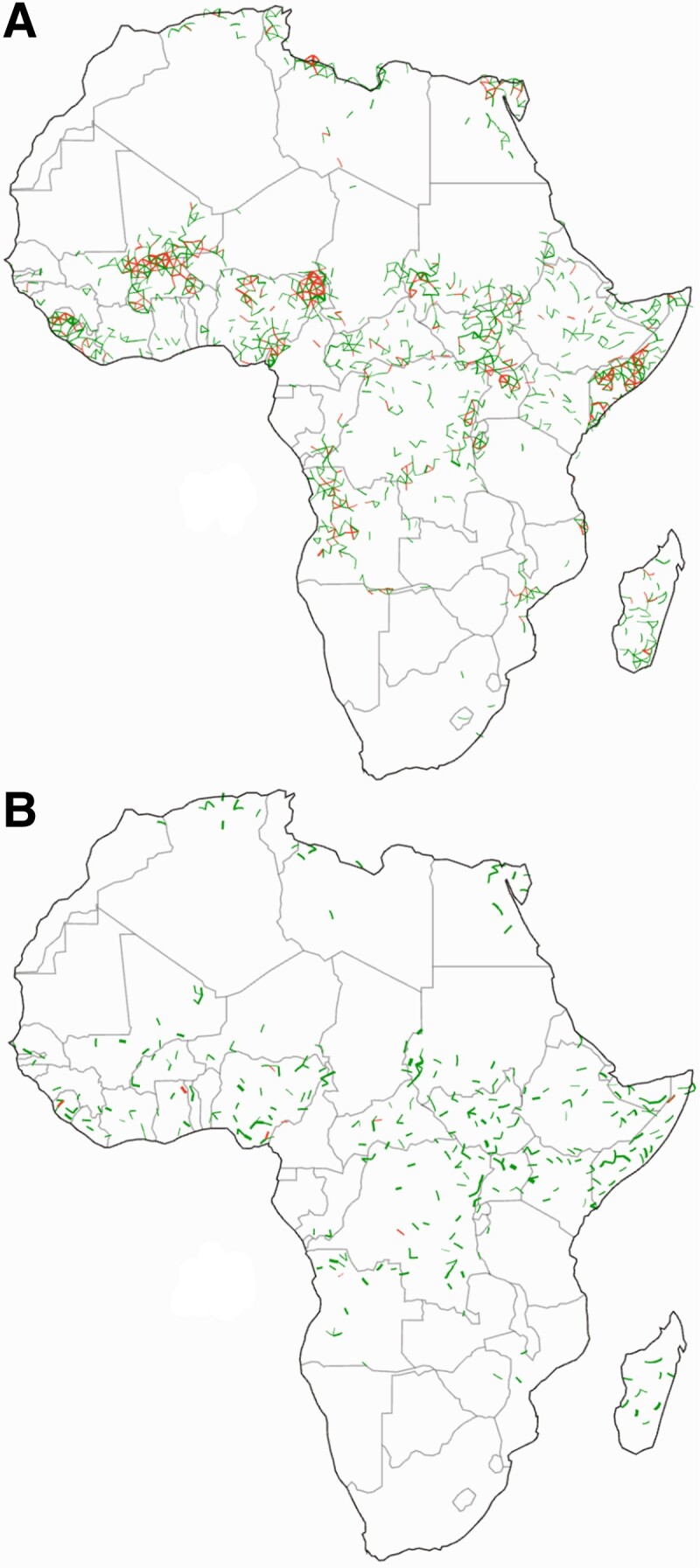
Causal network through which conflict avalanches propagate. A) Statistically significant causal edges between adjacent Voronoi cells using transfer entropy (a=64 days, b≈88 km). Directed nature of graph not shown. Edges shown in green have a causal edge in one direction only, red in both directions. B) Causal network from time-shuffled null model is fragmented.

The resulting “causal network” gives us a way of tracing chains of conflict events (see Fig. S2 to observe the networks derived utilizing alternative measures to transfer entropy). We connect all events to one another that have occurred together in the same spatiotemporal bin and that have occurred in any adjacent spatial bin at a sequential time to which there is an outgoing causal edge (see Appendix B and Fig. S1 for detailed algorithm). From such a procedure we obtain clusters that cascade over time, or *conflict avalanches* (see movie in reference 36), across a wide range of scales as we vary *b* between 10 to $10^{3}$ km (Africa is about $10^{4}$ km wide) and *a* from 1 day to $10^{3}$ days. As one picture of conflict avalanche extent, we color the geographic regions that a conflict avalanche covers in totality, joining together regions when avalanches intersect with one another to define *conflict zones*. These are the colored regions in Fig. 4C. We find that at the largest separation scales nearly all of Africa is lumped together into a single large conflict zone (top left map in panel A), whereas at the smallest scales Africa fragments into small disparate zones (bottom right map panel in panel B). Only in between the extremes do we find conflict avalanches covering a wide range of scales, displaying scaling statistics, and whose spatial extents are qualitatively recognizable as in Fig. 4C. This suggests the existence of some mesoscale at which conflict avalanches correspond to meaningful narratives of cause and effect.

**Fig. 4.**
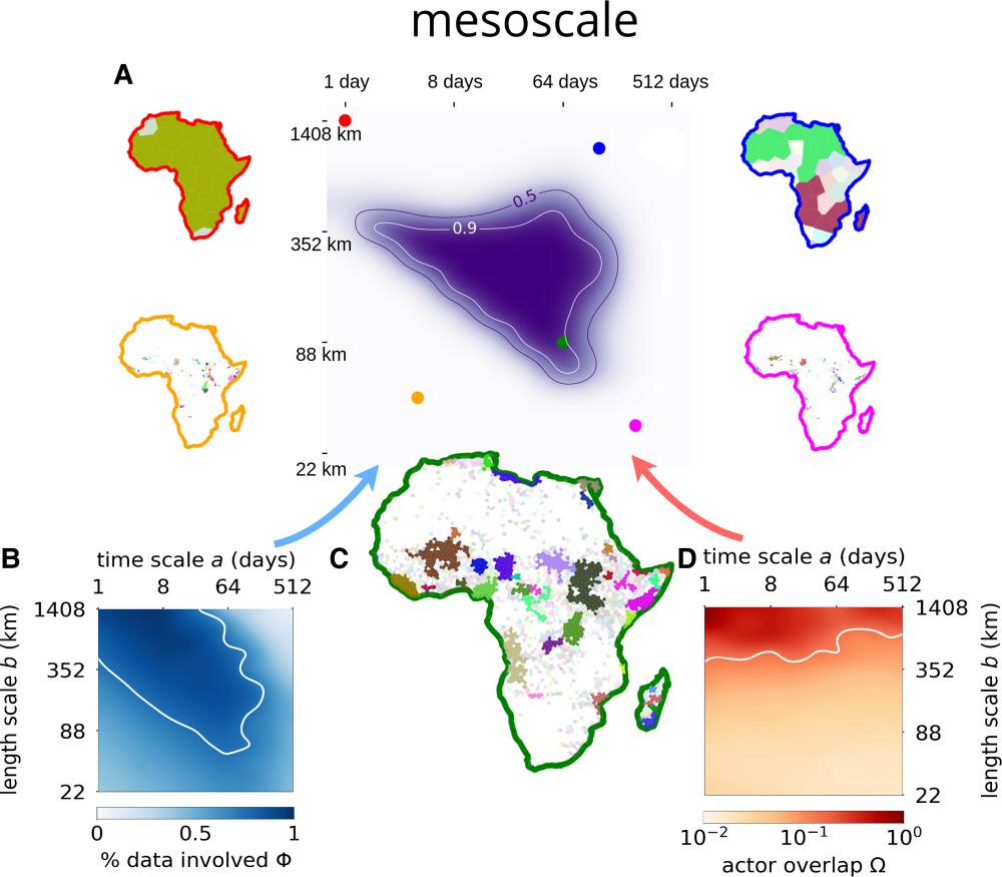
Mesoscale for armed conflict. A) Mesoscale is identified using scales in which conflict avalanches B) contain more than 3/4 of the data Φ≥3/4 and D) actor overlap is less than the midpoint of actor overlap score Ω≲0.132±0.002, with standard deviation given over Voronoi tessellations. (For hyperparameter (Φ,Ω) sensitivity analysis, see Figs. S12 and S13.) C) Example of conflict zones in the mesoscale, a=64 days, b≈88 km. The mesoscale in A) is obtained using an overlap of 100 different realizations of Voronoi tessellations (for details see Appendix I) and the contour lines show the regions which were in 50 and 90% of the tested mesoscales. For a few examples, we display the resulting conflict zones as indicated by the markers in panel A, where color corresponds to the outline of the respective African map. Colors inside each conflict zone map correspond to different zones. Conflict zones which span less than five Voronoi cells are shown but faintly.

To make this intuition more concrete, we propose a simple first-principles argument for isolating a mesoscale. First, we stipulate that most conflict events should belong in a conflict avalanche; otherwise, conflict avalanches are not a useful representation of the data. Since majority could mean anywhere from half to all of the data, the minimal choice, or the uniform prior, is the midpoint at Φ=3/4, or that at least that fraction Φ of the data must belong in a conflict avalanche. As we show in Fig. 4B, this threshold (the white line) delineates a region of scales in the upper left portion of the full space.

Second, we remark that the largest conflict avalanches tend to group disparate actors together even though conflict actors tend to be geographically localized. We quantify this intuitive criterion by defining an actor overlap Ω, which gauges how similar sets of actors are amongst all conflict zones. A standard metric would be to consider the normalized overlap between sets of actors between zones, but this fails to account for the possibility that some actors overwhelmingly dominate the set of observed events, whereas others may only appear once or twice. To account for this imbalance, we compute a weighted overlap that accounts for the fraction of events in which actors are involved for each pair of conflict zones (see Appendix G and Fig. S9). Our weighted overlap is the average over pairwise comparisons including self comparisons (where the weighted overlap is 1) such that Ω=0 when none of the conflict zones have overlapping actors, and it saturates at Ω=1 when all conflict zones have the same actor distribution. Therefore, actor overlap provides a normalized measure that accounts for how homogeneous or heterogeneous conflict zones are from one another as we show in Fig. 4D.

As before, we choose a minimally informed threshold for actor overlap. Continent-encompassing conflict zones show maximal overlap because they unite nearly all actors into a single large conflict, whereas fragmented zones fail to connect events perpetrated by the same actor. Absent other information, the minimal choice over the interval is the midpoint of overlap, which on a logarithmic scale is Ω≈0.132.^a^ In agreement with the observation that actors are mostly geographically localized, we show in Fig. 4D that this threshold cuts almost horizontally across at a fixed value of b≈350 km. Putting the two thresholds together (for hyperparameter (Φ,Ω) sensitivity analysis see, Figs. S12 and S13), we obtain in the intersection a *mesoscale* that we highlight in Fig. 4A, which denotes the region, derived from first-principles, where we anticipate nontrivial examples of conflict to be located.

The boundaries of the mesoscale represent a tradeoff between the spatial and temporal scales of analysis. Its shape indicates that at sufficiently short temporal scales of a few days to a week only a very limited range of geographic scales reveal identifiable and meaningful causal patterns. As we increase the temporal scale to about a month to a few months, however, a much wider window of spatial scales display widespread causal spatial dynamics, suggesting a more fruitful region of study as opposed to other timescales. All together, the spatial scales of interest are limited to between tens and a few hundred kilometers. This indicates that for practical purposes analysis of conflict spread is limited to a range of scales that span from b≈60 km to b≈400 km. To get a sense of what such extracted scales represent, we calculate the typical distance between neighboring populations using “urban agglomerations,” a generalized definition of a city as defined in the Africapolis data set (37), and we find the nearest neighbors for the smallest agglomerations are about ∼20 km in 2015, which is far below our lower cutoff (see Fig. S8). Only once we consider agglomerations with at least $10^{5}$ people do we find that the typical distance coincides with the minimum of our mesoscale at b≈60 km. The maximum of the mesoscale at b≈400 km aligns with the distance between pairs of large cities, or at least $10^{6}$ people. Thus, our method of extracting a mesoscale suggests that large population centers are what typically mediate conflict spread.

A notable feature of the mesoscale is that the conflict avalanches display a range of temporal and structural scales, or non-Gaussian statistics. Motivated by previous work (22), we compute power law fits to approximate the distributions of the avalanche properties for each combination of separation scales *b* and *a*: size in terms of fatalities and reports, geographic extent in terms of area and diameter, and duration, examples of which are shown in Fig. 5A–E. For any of these properties *X*, a probability distribution P(X) takes a power law form $P(X)∼X^{-\alpha }$ with positive exponent α but only above a lower cutoff $X\geq X_{\min}$. We find the fit parameters using a standard procedure (38). The majority of scales included in the mesoscale are consistent with power law tails and that the power law is often a better fit than a reasonable alternative model, the lognormal (see Table S1), by the likelihood ratio in the tail of the distribution (see Appendix E, Figs. S6 and S7 for more details). The power law tails in the mesoscale indicate that, beyond some minimal size, conflict avalanches display multiple relevant scales for dynamics and size, consistent with a mesoscale that spans a range of scales.

**Fig. 5.**
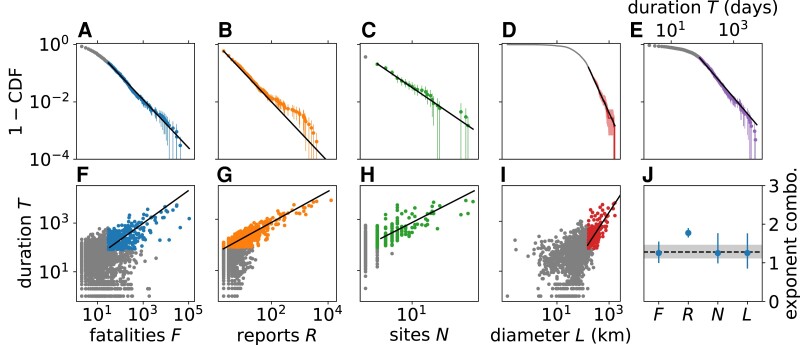
Evidence of long-range correlations in the mesoscale for b=176 km, a=64 days. A–E) Distributions of avalanche properties have exponents τ=1.8±0.1 (p=0.73), $\tau^{\prime }=2.07\pm 0.03$ (p=0.002), μ=2.7±0.4 (p=0.56), ν=3.0±0.4 (p>0.99), α=2.3±0.1 (p=0.075). A *p*-value of greater than 0.1 is significant, which indicates that some of the fits are only approximately power laws (38). Points below the lower cutoff are gray. Exponent error bars represent one standard deviation over $10^{3}$ bootstrapped samples. Shown error bars in plan correspond to 95% confidence intervals over the same bootstrapped samples. F–I) Duration vs. conflict measures, or dynamical scaling. Points below the respective lower cutoffs in the power law distributions are not fit and are shown in gray. J) Predicted exponent relations relating exponent for duration distribution α (shaded region) vs. exponent combination for remaining variables (markers) align for all except reports *R*, which deviates from a power law distribution. This is an indication of yet unexplained mediating variables or processes missing in the dimensional analysis.

Just as length and volume are related in a fixed way for physical objects, conflict properties represent different dimensions that are quantitatively connected. We relate them by noting that we typically expect longer conflicts to become larger. This can be expressed as a dynamical scaling hypothesis for fatalities *F* with duration *T*, or that $F∼T^{d_{F}/z}$ for a positive exponent $d_{F}/z$.^b^ Then, we predict $\tau -1=d_{F}(\alpha -1)/z$ for the power law model distributions $P(F)∼F^{-\tau }$ and $P(T)∼T^{-\alpha }$. A similar relation can be derived for each of the other properties. The exponent relations are a predictive test and are usually satisfied in the mesoscale for fatalities, sites, and diameter but not for reports (Fig. 5J). Reports aside, the exponent relations indicate that most aspects of conflict avalanches conform approximately to a low-dimensional theory at sufficiently large scales (3, 22, 39) (see Fig. S11). Thus, the scaling patterns, by revealing nonlocal structure, are a first confirmation that our causal network tracks complex and dynamic objects.

As further validation that goes beyond the statistical patterns, we check if conflict avalanches coincide with causal mechanism identified in the conflict literature. As we show in Fig. 6, identifying conflict clusters by the names of involved actors in Nigeria leads to four major, spatially overlapping conflict clusters for Boko Haram (red), Fulani militia (green), the People’s Democratic Party (orange), and Ambazonian separatists (blue). For a choice of separation scales that is comparable, example conflict avalanches in Fig. 6B group events differently. Some Fulani militia attacks south of the red Boko Haram cluster form part of the latter rather than a separate group as in panel A. Our clustering is supported by field studies, where it has been pointed out that clashes between Boko Haram and local herders drive the latter further from their normal ranges in northeastern Nigerian leading to conflict between herders and farmers (18). Furthermore, we identify the events associated with the conflict at the border of Nigeria and Cameroon as green in panel C, which are generally unrelated to the purple events in the northwest. Taken together, this is consistent with the “Triangle of Terror” in Nigeria (40): Boko Haram (red), Fulani Militia and Anglophone crisis (green), and banditry prevalent in the Zamfara region (purple) (41). This particular example confirms that we are able to extract clusters that qualitatively correspond to but also could enhance studied conflict groupings.

**Fig. 6.**
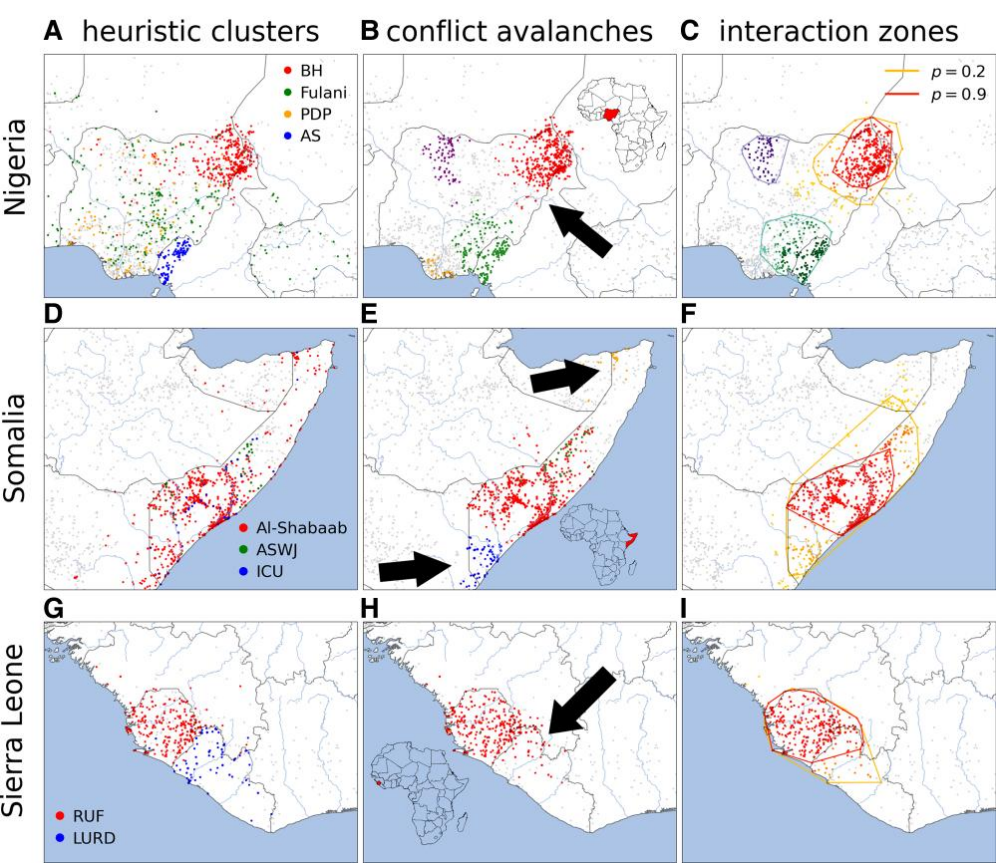
Heuristic conflict clusters vs. our systematic conflict avalanches. Light points in background (gray) represent all the conflict events that are not part of the shown conflict clusters and avalanches. Conflict in A–C) Nigeria with b≈88 km, a=64 days, D–F) Somalia with b≈88 km, a=32 days, and G–I) Sierra Leone with b≈66 km, a=64 days. Scales have been chosen to align with mechanism from field studies. A,D,G) Conflicts identified by actor names. B,E,H) Geographically biggest conflict avalanches in the shown region sampled from the pseudorandom Voronoi realizations are representative of C,F,I) conflict interaction zones incorporating the probability p that events belong to the highlighted conflict avalanche; inner hull is of high confidence and outer hull is of low confidence. Arrows point to conflict events that are associated with B) Fulani Militia but are grouped with the Boko Haram cluster in A,E) Al-Shabaab attacks which are separate from the central Al-Shabaab cluster in red, and H) LURD events combined with RUF. (C) Top left (purple) and bottom (green) convex hulls correspond to p=0.5. To see the variation in conflict avalanches and interaction zones across the mesoscale see Figs. S14, S15. Acronyms as follows: BH, Boko Haram; PDP, People’s Democratic Party; AS, Ambazonian Separatists; ASWJ, Ahlu Sunna Waljama’a; ICU, Islamic Courts Union; RUF, Revolutionary United Front; LURD, Liberians United for Reconciliation and Democracy.

As a more systematic look, we can look across the many instances of conflict avalanches that are produced from our algorithm, the exact details of which vary with the randomness in the Voronoi tessellation. By averaging over the different tilings, we measure the strength of the causal connection, or the probability *p* that any two events are joined into the same conflict avalanche. After calculating this probability for the central events in the Boko Haram avalanche (which always appear together), we draw the convex hulls containing the outermost points for fixed values of *p* in Fig. 6C. These are regions of causal interaction that confirm the core of Boko Haram insurgency that is always grouped together as indicated in red (to see the variation in conflict avalanches and interaction zones across the mesoscale see Figs. S14, S15). Further out, the regions reveal a substantially stronger relationship between the core and the events involving the Fulani militia. For comparison, we also show the p=1/2 contours for Zamfaran banditry and Ambazonian rebels. Importantly, repeating this exercise across other examples reveals that causal interaction is not simply a function of geographic distance, but events in the same location can trace distinct causal origins. Remarkably, our approach relying only on statistical proxies for causality discovers causal mechanism hypothesized in field and conflict studies, suggesting that this provides a powerful scope for identifying hidden interactions.

For two other examples, we inspect conflict in Somalia and Sierra Leone. In the case of Somalia, we show that different scales reveal underlying structure in the local and regional components of the different subgroups of the Al-Shabaab insurgency. As an example, the bottom arrow in Fig. 6E points to conflict events associated with Al-Shabaab which are not clustered with the northern (red) core. Indeed, the former were not part of the initial insurgency and instead caused by conflict with Kenya (blue) when Kenya invaded southern Somalia to “flush-out” Al-Shabaab (42). Similarly, the top arrow points at a local chain of violence around Bosaso due to the presence of Al-Shabaab and its support groups in the area. This is reflected in the regions of causal interaction that reveal a core of high confidence p≥0.9 along the Shebelle river in the south, which is only weakly linked with events in the north. In Sierra Leone, our clustering procedure strongly suggests (p≥0.9) that the events perpetrated by the Revolutionary United Front (RUF) are related to those in Liberia by Liberians United for Reconciliation and Democracy (LURD). Such a relationship is purported by court allegations that Sierra Leone’s government helped in the formation of LURD by training fighters in Guinea (43) and further substantiated by RUF fighters joining LURD (44). Another mechanism is alleged fights between RUF supporters and LURD opponents of warlord Charles Taylor in Liberia’s Lofa county (45). In contrast, events in Côte D’Ivoire are highly unlikely to be related with p≤0.1 as the dearth of literature on the relation between the two conflicts suggests. Thus, our approach provides a systematic way of measuring area of causal interaction across local to regional scales with a natural measure of uncertainty to mine or to disprove causal relationships.

## Discussion

All conflicts have multiple narrative scales, which can range from the detailed role of the individual (the assassination of Archduke Franz Ferdinand instigating World War I) to geopolitics (a secret alliance network consequently implicating many nation-states (2)) and even further out to societal epochs across civilizational timescales (46, 47). Other narratives range from the role of ideologies, personalities, economic incentives, organizational resources and structure, etc. (see references cited in 8, 19). Each narrative implicitly assumes a relevant range of scales over which to draw a causal relationship between intervening events. And the quantitative evidence confirms that multiple scales matter: many conflict patterns beyond a small size are scale-free, which means that no single scale holds a privileged perspective (22, 48–51). This points to a fundamental challenge in the study of armed conflict, which is that conflict consists of many events occurring at multiple, overlapping spatial and temporal scales (5, 6). As a result, methods for clustering conflict events must incorporate an adjustable scale in order to engage with the full complexity of conflict (52).

We develop a systematic, data-driven, and scale-dependent procedure for extracting chains of causal events, or “conflict avalanches,” from observational data that could serve as the basic objects of conflict study (Fig. 2). We construct conflict avalanches using a filter for statistical signatures of causality with a general measure of predictability, transfer entropy, which is a widely used measure for identifying hidden connections between system components (24, 26, 53). Here, we use transfer entropy to build causal networks that connect local conflict events to one another. To do so, we start with the assumption that causal patterns can be detected from a reduced time series that only considers the appearance or absence of conflict, or binarization that ignores the magnitude of events. On one hand, this is a practical solution for handling general challenges in estimating statistics from a large state space. On the other hand, the power of the simplification is borne out in how we successfully identify related events, meaningful scales, and causal mechanisms hypothesized in the literature.

We discover a mesoscale at which conflict avalanches align with sociopolitical intuition (Fig. 4), corresponding to separation scales on the order of a few days to months and tens of kilometers to hundreds. First, we recognize that the geographic scales recovered *a priori* range from 60 to 400 km. Reassuringly, this is the typical distance between large neighboring towns and cities, which are important geographic pinning points for conflict (see Appendix F). The lower cutoff is much larger than any individual urban agglomeration and implies that conflict relations are not visible at microscopic precision. This could be because there is truly little statistical signal at such level of detail or from the limited resolution of the ACLED data set. Furthermore, the irregular shape of the mesoscale indicates that space and time scales are not independent of one another, or that looking at longer time scales is not equivalent to looking at longer spatial scales. Finally, we find that the avalanches in the mesoscale display a wide range of dynamical and spatial structures such as power law scaling. Such patterns indicate that conflict avalanches reflect long-range correlations between events. Thus, the mesoscale presents an interesting set of scales in which to focus on causal conflict patterns.

Perhaps surprisingly, conflict avalanches in the mesoscale group together events in a way that aligns with causal mechanism proposed in the literature. We compare our conflict avalanches with heavily studied conflicts in Eastern Nigeria, Somalia, and Sierra Leone. In each of the cases, our method recovers recognizable clusters of events that align with actor groups and distinct phases of conflict. Yet, we also find surprising connections when we draw regions of causal interaction. With Nigeria, we connect with non-negligible probability events that are identified as Fulani militia with the Boko Haram core, suggesting that these conflicts are related to one another. In Sierra Leone, we connect RUF government forces with events in neighboring Liberia, in line with allegations of troops crossing the border. For these examples of causal validation, we focus on relatively short scales at the bottom corner of the mesoscale, but at larger geographic scales we also discover causal influence regions that highlight regional conflict patterns (54) (see Fig. S10). This suggests that beyond confirming known cases of causal relationships, our procedure can provide a way of predicting new ones to test, refine, or inspire new hypotheses.

As a step in this direction, we develop conflict zones of causal interaction (Fig. 6). The zones are convex hulls of the probability that a nearby event is grouped into a conflict avalanche with the seed events. This is a practical application of our work to a problem that has attracted much attention in the literature (4, 11). It could, when coupled with expertize in the particular conflict zone of interest, enable better policy decisions and the impact of conflict on related phenomena such as poverty, segregation, and crime (11). Importantly, we introduce the flexibility of an adjustable spatiotemporal scale which can be crucial for detecting patterns that only emerge at certain levels of coarseness (6, 55–58). For example, the relationship between rainfall variability and conflicts is indiscernible when using a large temporal window such as an year as compared to monthly temporal window (59). Conflicts show correlation with climate-related disasters only when the period of analysis is less than three months (60), and more generally the choice of scale is important for the connection between climate change and conflict (61). Poverty and conflict is most meaningful at the subcountry level (12). Our scale-adjustable scope provides a natural way of handling such variability to identify areas of potential interest or highlight unseen connections that deserve deeper investigation.

The need to bridge microscopic and macroscopic descriptions generalizes to other spreading social processes including unrest, migration, epidemics, and their relationship to conflict. As such, our approach has potential for wider use. For example, estimates of the eventual geographic extent of new activity can inform response planning. Our minimal approach may be especially helpful in this regard because it is difficult to gather detailed and accurate information in conflict regions (62). As another example, event avalanches can feed into automated methods of pattern discovery by providing structured input for training machine learning algorithms (52, 63–65). Our event avalanches can provide groupings across a hierarchy of scales, which can be further enhanced by other properties that we have not considered here like sociodemographic factors. Thus, we address a fundamental need for a tool in both policy and quantitative analysis for handling multiscale social processes, and we pave the way to explore, rather than be limited by, the variability across scales.

## Supplementary Material

pgad228_Supplementary_Data

## Data Availability

Code for this article can be found at the Github repository. Voronoi tessellations and conflict avalanches generated for Africa between the period 1997 and 2020 are available at the Zenodo repository, DOI: 10.5281/zenodo.8117567.

## Notes

a The logarithm accounts for the fact that similarity *S* is heavily skewed towards 0, when we have many avalanches and most pairwise comparison consist of unrelated regions.

b The exponent is written in this way because $d_{F}$ is known as the fractal dimension and *z* the dynamical exponent.
