# Supplementary material for: Discovering the mesoscale for chains of conflict
Source: PNAS Nexus. 2023 Aug 1;2(7):pgad228. doi: 10.1093/pnasnexus/pgad228 (PMC10392960; doi:10.1093/pnasnexus/pgad228)
Supplement: pgad228_Supplementary_Data [file pgad228_supplementary_data.pdf]

Supplemental Information

A. Dataset

Our primary dataset is the Armed Conflict Location & Event Data (ACLED) Project. This project collects data on armed conflicts around the world with a focus on African states. The dataset is a collection of individual conflict events, defined as a single incidence of violence at a particular location and time involving at least two actors. In our analysis, we primarily focus on the location and date of the conflict events, and we use other information including actor identities and event description for validation of the conflict avalanches.

Other event-based armed conflict datasets besides ACLED include the Global Terrorism Database (GTD); the Integrated Crisis Early Warning System (ICEWS) dataset; the Phoenix event dataset; the Global Database of Events, Language, and Tone (GDELT); and the Uppsala Conflict Data Programme Georeferenced Event Dataset (UCDP GED) (1). We choose to use ACLED in our analysis because of two major reasons:

1. Event-based armed conflict databases extract their information from various news reports from multiple sources. This can be done either manually by the help of human researchers and experts or can be scraped automatically from news articles. Since we are focusing on Africa, we require a dataset which is curated manually by experts since most news articles published in Africa are not in English and should have some understanding of local context. ACLED, GTD, and UCDP GED are the only three expert-curated datasets. The others are compiled using automated systems which tend to be heavily biased towards conflict events reported in English and French media (1) since currently automated systems are not designed to crawl through local language media.

2. ACLED covers all violent activities that occur both within and outside the context of a civil war, particularly violence against civilians, militia interactions, communal conflict, and rioting. The other data sets do not. GTD focuses on "terrorism" only. UCDP GED only records conflict events with at least one fatality. These definitions of armed conflicts are too restrictive for our purposes. Therefore, ACLED is the most suitable dataset for our analysis among the available event-based datasets.

In ACLED, the conflict events are categorized into five major types. We mainly focus our analysis on conflict events that are categorized as "Battles." According to the ACLED codebook, there are three different kinds of battles that we include in our Battles conflict avalanches. As quoted from the codebook, these are defined as follows:

1. Battles - No change of territory: "A battle between two violent armed groups where control of the contested location does not change. This is the correct event type if the government controls an area, fights with rebels and wins; if rebels control a location and maintain control after fighting with government forces; or if two militia groups are fighting. Battles take place between a range of actors."

2. Battle - Non-state actor overtakes territory: "A battle between two violent armed groups where non-state actors win control of a location. If, after fighting with another force, a non-state group acquires control, or if two non-state groups fight and the group that did not begin with control acquires it, this is the correct event. There are few cases where opposition groups other than rebels acquire territory."

3. Battle - Government regains territory: "A battle between two violent armed groups where the government (or its affiliates) regains control of a location. This event type is used solely for government re-acquisition of control. A small number of events of this type include militias operating on behalf of the government to regain territory outside of areas of a government's direct control (for example, proxy militias in Somalia which hold territory independently but are allied with the Federal Government)."

DRAFT

B. Algorithm for generating conflict avalanches

We devise a systematic method to generate conflict avalanches for different levels of resolution, which are indexed by combinations of spatial and temporal scales.

- **Choice of scale**

  To set the spatial scale, we divide Africa into Voronoi cells of approximately equal area. To do this, we generate a tiling using a Poisson disc-sampling algorithm (2, 3). The spatial scale is set by setting the average distance between the centers of two neighboring cells to be approximately b km. To set temporal scale, we divide the total number of days in the dataset into contiguous sequence of bins of duration a days. One spatial cell indexed x and temporal bin indexed t together form a spatiotemporal coordinate system for conflict events (Figure S1c).

- **Binarization**

  We label a spatiotemporal cell with at least one conflict event as active; otherwise, it is inactive. This procedure returns a binary time series in each Voronoi cell (see movies in references 4 and 5).

- **Causal network**

  We search for causal relationships between neighboring spatial cells from the statistics of conflict activity with the transfer entropy (Eq 2). If the value of transfer entropy between a pair of cells is significant (with $p \leq 1/20$) compared to a time-shuffled null model that erases temporal ordering of events in each cell, we detect a causal link between two cells. Since transfer entropy is asymmetric, we must calculate it in both directions for every pair. If the magnitude of transfer entropy is significant in both directions, we get a bi-directional causal

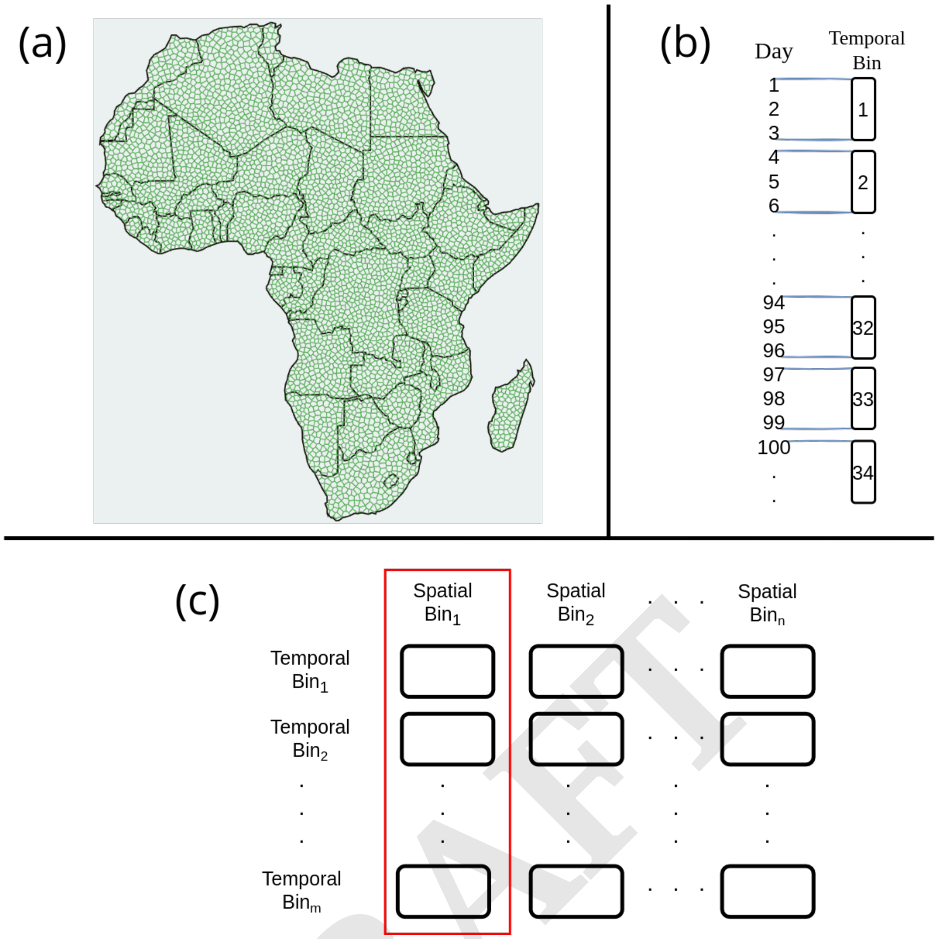

Fig. S1. (a) Spatial Voronoi cells. $b \approx 88$ km. (b) Temporal bins. In this example the temporal bins are of size $a = 3$ days. (c) Spatiotemporal bins. Each box here is a spatiotemporal bin. Each spatiotemporal bin can have one of two values, one or zero. One represents presence of conflict and zero represents absence. Every spatiotemporal bin which has value one is called a "packet" of conflict event(s). Each column here is a binary time series of an individual spatial cell. For example, the spatiotemporal bins in the red box forms the time series for spatial bin number 1.

DRAFT

link as we show in Figure 4. If the magnitude is significant only in one direction, we get a uni-directional causal link. We do this for all adjacent pairs of cells to construct a causal network.

We search for a self-causal loop (an edge from a spatial cell at time t to itself at time $t+1$) using the transfer entropy, which reduces to the mutual information in Eq 1.

- **Clustering events**

  We cluster together every pair of conflict events that satisfies one of the three following conditions:

  – The events occur in the same spatiotemporal bin (x, t).

  – The events are sequential in time bin and belong to the same cell with a self loop.

  – The first event occurs at time t and cell x, and the second occurs at time $t+1$ and another tile x'. There is a causal edge from x to x'.

  Once every pair has been clustered (some will remain alone), we have conflict avalanches.

C. Other ways of inferring causal network

In principle, one could have constructed the "causal" network using other measures of temporal predictability such as Granger causality, time-delayed correlation, and time-delayed mutual information. We do not consider Granger causality because the variables we consider are not Gaussian, the assumption underlying that measure. While the latter measures do not explicitly distinguish the directionality of time because they are time-symmetric, we can still measure asymmetric information between sites by testing one site to be the past of the other and vice versa.

We show in Figure S2 the resulting networks from the time-delayed pairwise correlation in panel a and with time-delayed mutual information in panel b. In the same way as with the transfer entropy calculation, we flag an edge as significant if it is of higher value than 95% of bootstrapped time-shuffles. Unsurprisingly, the alternative measures return dense concentrations of links in similar areas as with the transfer entropy. In contrast, networks are denser both within the conflict hotspots and in more remote regions, suggesting that the transfer entropy provides a more discriminatory approach on which to build conflict avalanches.

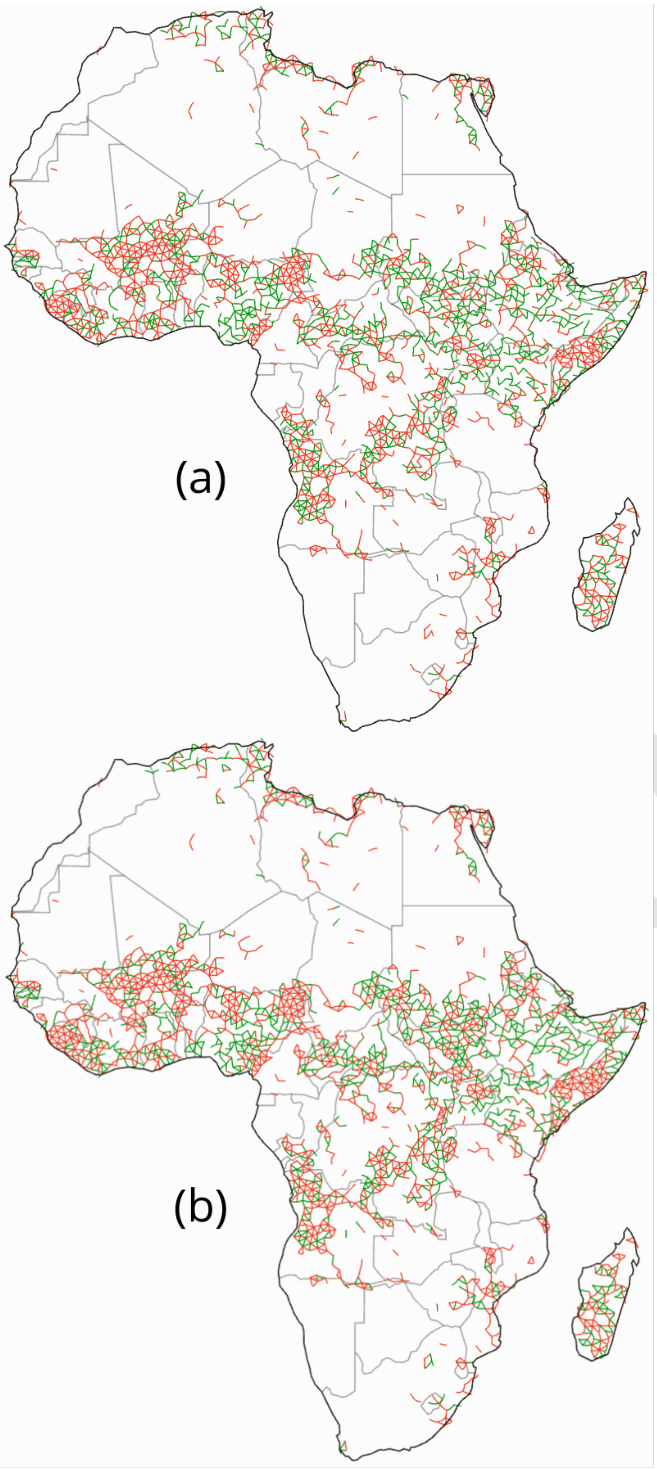

Fig. S2. Conflict network inferred using (a) time-delayed pairwise correlation and (b) time delayed mutual information for $a = 64\,$days, $b \approx 88\,$km. We use 95% bootstrapped confidence intervals to identify significant links between neighboring spatial bins. Directed nature of graph not shown. Edges shown in green have a causal edge in one direction only, red in both directions.

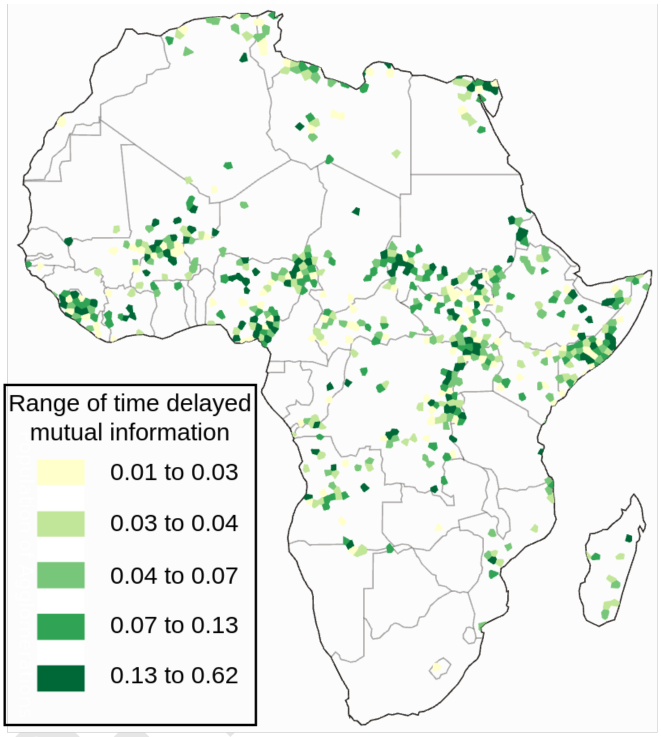

Fig. S3. Time-delayed mutual information (Eq 1) at Voronoi cells ($a = 64\,$days, $b \approx 88\,$km).

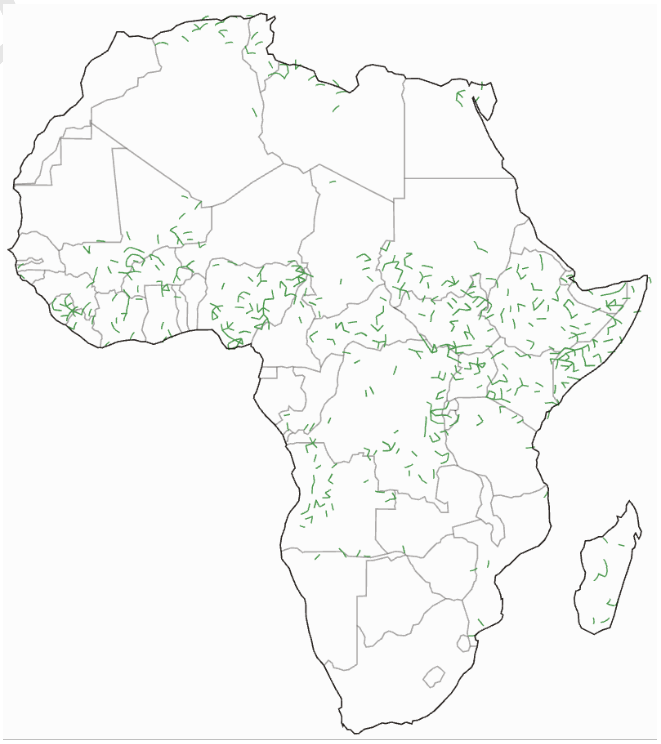

Fig. S4. Causal network for space-shuffled null model. Temporal scale $a = 64\,$days and spatial scale $b \approx 88\,$km.

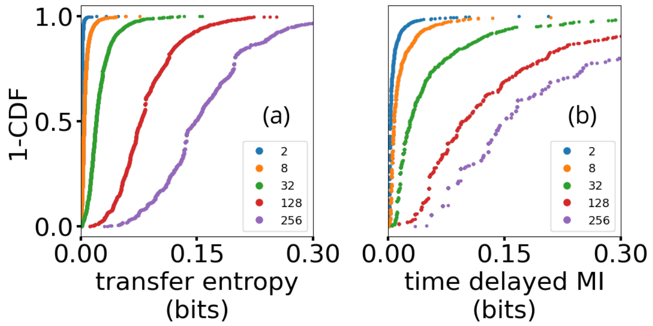

Fig. S5. Complementary cumulative distribution function of (a) transfer entropy values of all valid links in a causal network and (b) mutual information between past and future of all self inciting spatial bins. Spatial scale $b \approx 88$ km and different colors correspond to different temporal scale a.

D. Null models

To test the procedure for extracting causal networks, we compare the causal network from data with two different randomized null models.

The first null model is time-shuffled, where we randomly reshuffle the binary sequence of activity in every single Voronoi cell independently of one another. This removes all temporal correlations and thus renders the Voronoi cells independent of one another. When we perform the same causal network construction procedure on the time-shuffled network, we still identify a few edges as significant because the p-value threshold will yield false positives. However, the network is structurally different from what we show in Figure 4a because it fragments as we show in Figure 4b.

The second null model is space-shuffled, where we reassign randomly the entire temporal sequence of activity from one Voronoi cell to another. We only do this for cells that have at least one conflict event recorded. Again, the causal network fragments as we show in Figure S4.

That the null models lose the interesting features such as the dense locales of intertwined conflict that otherwise appears in the data confirms that our results are not artifacts of the procedure.

E. Modeling the distributions of conflict properties

We measure several properties that characterize conflict avalanches including the number of reports R, fatalities F, duration T, diameter L, and area measured by the number of Voronoi cells N. When the properties are taken over the ensemble of avalanches, they are typically (though not always) consistent with power law tails in the mesoscale. In order to reach this conclusion, we compared the power law against a lognormal and exponential, which are alternative models for a heavy-tailed distribution and for the absence of a heavy tail, respectively. The probability distributions are defined in Table S1, and they all account for the fact that measurements of value 1 are anomalous for conflict avalanches as is pointed out in the supplementary information of previous work (6). For fatalities, this is a known data issue. Thus, we only consider each property of conflict avalanches with $F > 1$, $R > 1$, $T > 1$, or $N > 1$.

For each data point x_{k}, we compute the likelihood of having observed it given the statistical model of the distribution.

Table S1. Definitions of distributions used to fit conflict properties (7).

	Distribution	Equation $p(x) = cf(x)$: $f(x)$	c
Continuous	Power law	$x^{-\alpha}$	$(\alpha-1)x_{\mathrm{min}}^{\alpha-1}$
	Lognormal	$\frac{1}{x}\exp(-\frac{(\ln(x)-\mu)^2}{2\sigma^2})$	$\sqrt{\frac{2}{\pi\sigma^2}}\left[\mathrm{erfc}\left(\frac{\ln x_{\mathrm{min}}-\mu}{\sqrt{2}\sigma}\right)\right]^{-1}$
	Exponential	$\exp(-\lambda x)$	$\lambda\exp(\lambda x_{\mathrm{min}})$
Discrete	Power law	$x^{-\alpha}$	$\frac{1}{\zeta(\alpha, x_{\mathrm{min}})}$
	Exponential	$\exp(-\lambda x)$	$(1-\exp(-\lambda))\exp(\lambda x_{\mathrm{min}})$

Looking over all the data points, we obtain the likelihood of the set of data

$$L(\{x_{\mathrm{k}}\}) = \prod_{\mathrm{k}=1}^{K} p(x_{\mathrm{k}}), \qquad [1]$$

where k is an index over the K data points, and the probability according to the model is $p(x_{\mathrm{k}})$. Since the number of data points that we fit depend on the model — for example, the power law comes with a lower cutoff — we compute the typical log-likelihood for each data point that was fit by normalizing by the total number of data points considered K such that we have $\log L/K$.

In our procedure, we would like to verify if the tail of the distribution resembles a power law beyond what we might reasonably expect from a comparison with the null models. This goal indicates that we should compare the fit to the tail of the distribution conditioned to be above the power law's lower cutoff. As we show in Figure S6, we find that the power law is superior *in the tail* to lognormal distribution in much of the mesoscale. In each box, we indicate with the color the typical gain in log-likelihood we have with the power law model taken over the random Voronoi tessellations and with the number the fraction of times the power law is superior; we have excluded cases where the number of data points $K < 20$ and the range of the data fit to the power law ranges over less than a decade since the statistics become unreliable. The lognormal tends to be superior on for short time scales a (left side of graphs), but when it is overall better in the mesoscale the difference is slight. In Figure S7, we show the exponential is always worse. In this sense, we rely on the power law as a useful and approximate scaling hypothesis on which to relate the conflict properties to one another above some minimal scale.

F. Population density

For gaining intuition about how the separation scales b and a relate to other social and geographic factors, we look at a map of population centers from the data set Africapolis. Africapolis considers population centers to be an "urban agglomeration" if the population exceed 10^4 and there is no gap greater than 200 meters between built spaces (8). Population counts are extracted using census data and the built space is determined from satellite imagery. This provides a systematic and universal definition of a city, called an "urban agglomeration," which does not depend on the vagaries of country records, datasets, and administrative definitions.

DRAFT

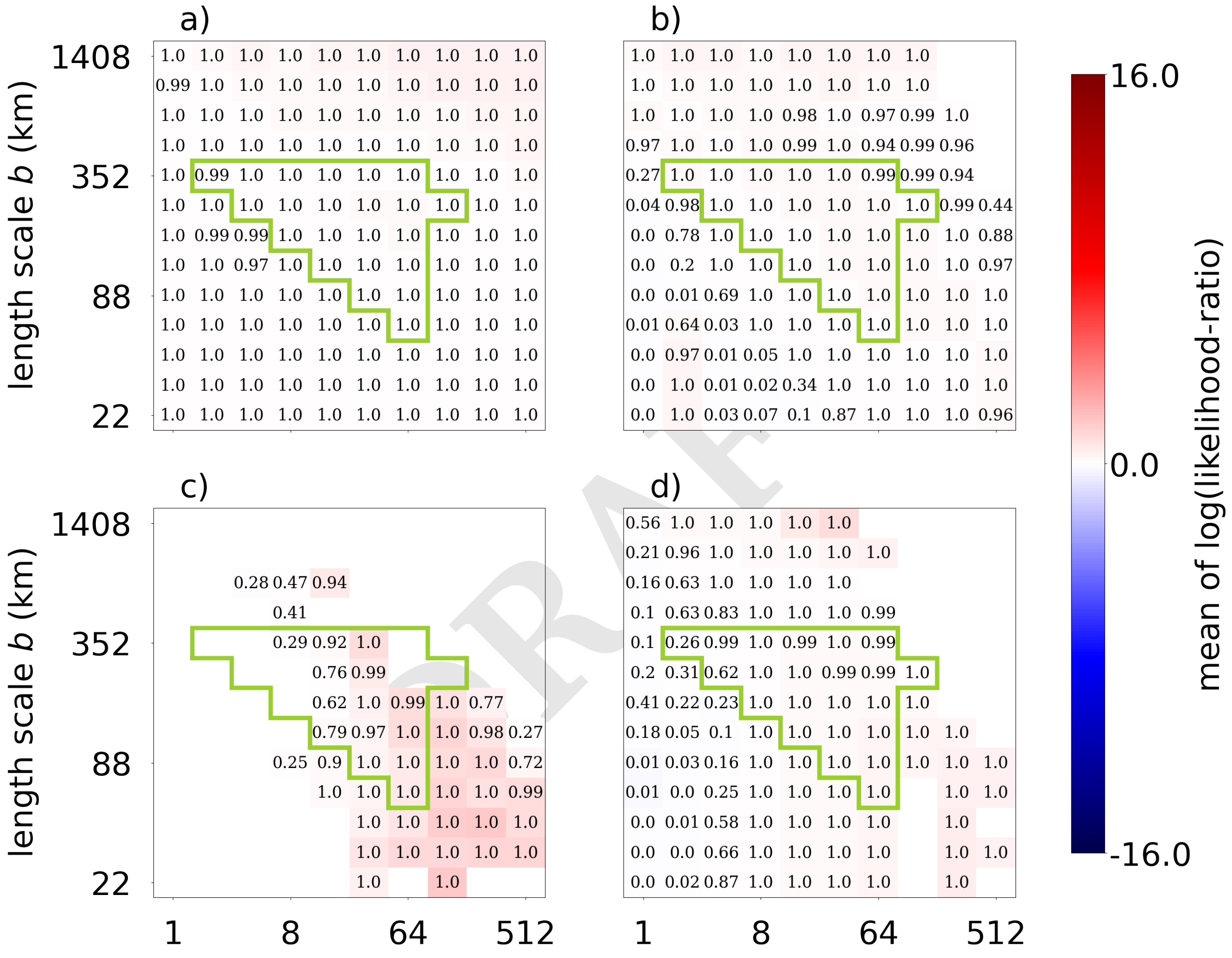

DRAFT

Fig. S6. Comparison between power law and lognormal distribution for (a) fatalities, (b) reports, (c) sites, (d) duration. Each block is centered at a spatiotemporal scale (see Appendix I for more details) at which we compare the log-likelihood of the two models. Green line encircles the mesoscale. Colors show the difference between log-likelihood of power law and lognormal distribution averaged over 100 pseudorandom Voronoi tessellations, i.e. red means that the power law is on average better. Number inside each spatiotemporal block shows the fraction of pseudorandom Voronoi tessellations for which power law is better. Not shown are scales with either less than 20 avalanches or range of less than a decade above the power law minimum.

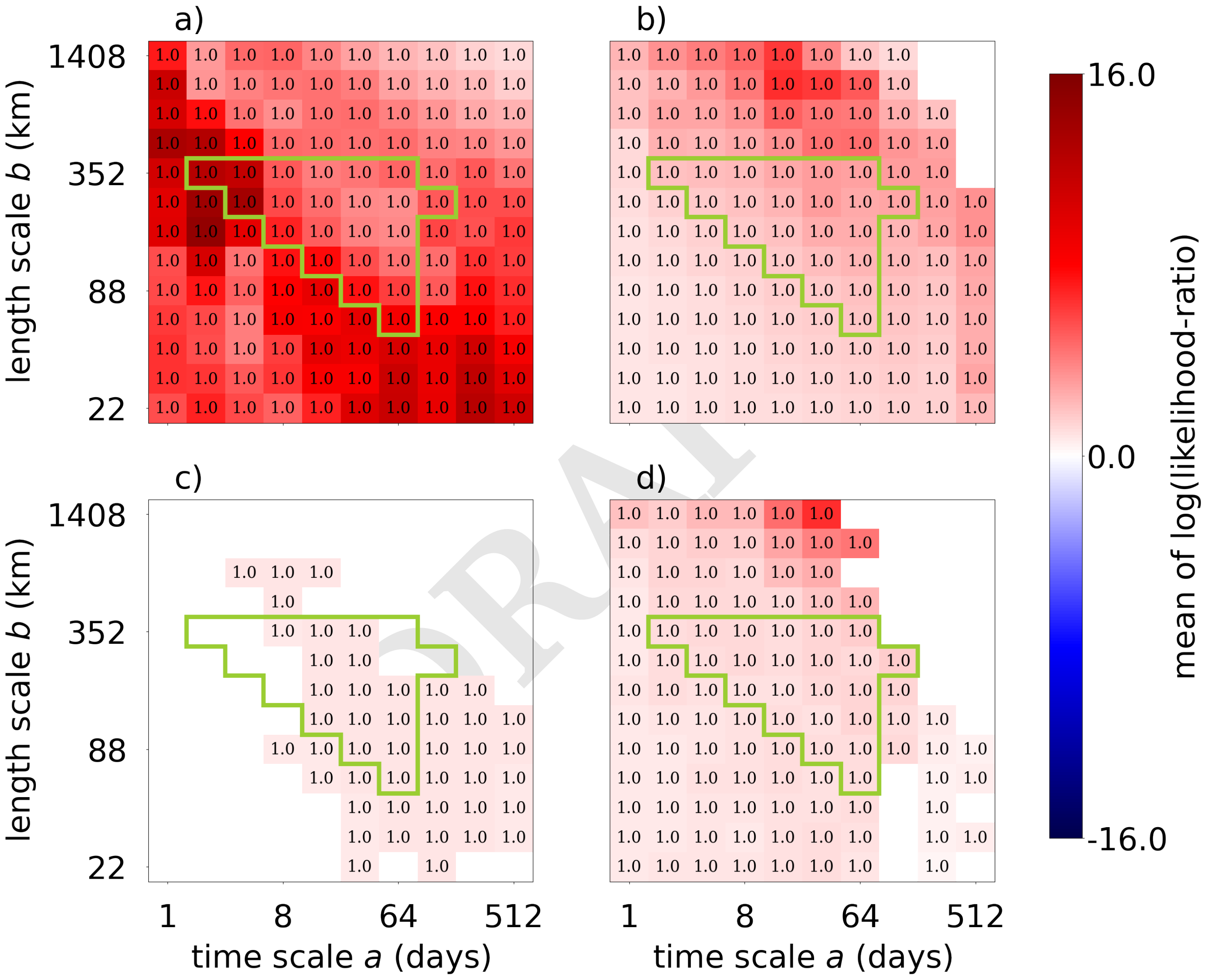

Fig. S7. Comparison between power law and exponential distribution as in Figure S6 for (a) fatalities, (b) reports, (c) sites, (d) duration.

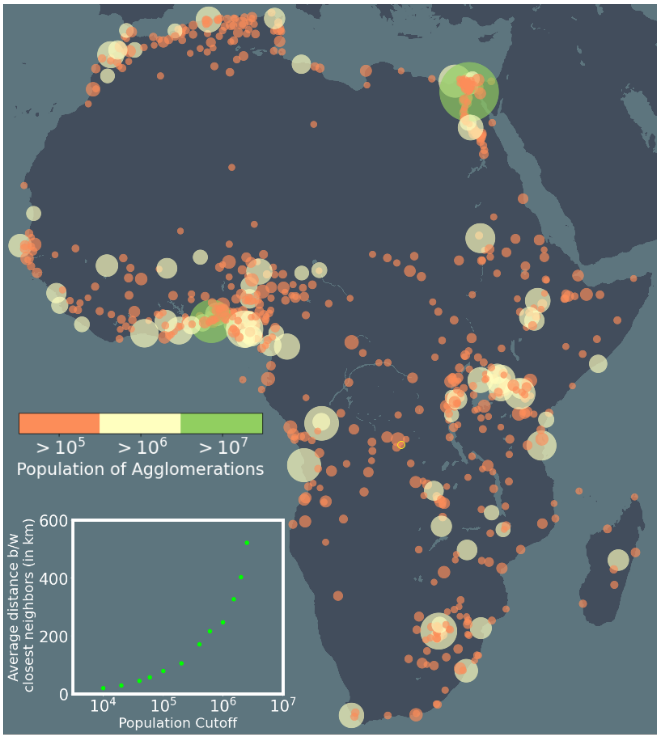

Fig. S8. Urban agglomerations in Africa according to the Africapolis dataset. The radii of the circles are proportional to the agglomeration's population.

As we discuss in the main text, we use Africapolis to extract distances between the centers of agglomerations. We find that the mesoscale at which causal conflict patterns emerge corresponds to a geographic distance of 60 km and 400 km, which is the typical distance separating nearest agglomerations of above 10^4 people and above 2,000,000, respectively. These average distances, however, may differ between regions. For example, East Africa shows larger distances between large agglomerations. This suggests that when focusing on regions of Africa particular sections of the conflict mesoscale may be more revealing than others. Indeed, we find that when we compare the conflict regions obtained in Nigeria (which a relatively dense region) with those from Somalia (a relatively sparse regions), we find that the relevant separation scales to be smaller in the former than the latter. While population density is not a perfect correlate of the amount of armed conflict, we find that population density matters for extracting causal relationships between conflict activity.

G. Actor overlap score

To calculate the actor overlap score Ω, we first construct an actor overlap matrix M comparing all pairs of conflict zones. Each element in the matrix accounts for the overlap between the sets of actors in each conflict zone weighted by the fraction of events in which they appear. In other words, each element $m_{\rm ij}$ of this matrix is calculated as the overlap between a pair of conflict zones indexed i and j,

$$m_{\rm ij} = \Theta_{Z_{\rm i}} \,.\, \Theta_{Z_{\rm j}} \qquad [2]$$

where $\Theta_{Z_{\rm i}}$ is a vector with each entry as the fraction of events in zone $Z_{\rm i}$ that involve each actor. All the diagonal elements

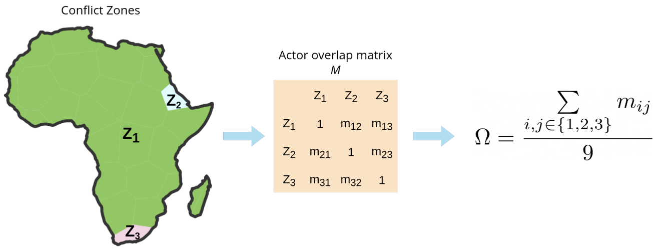

Fig. S9. Visual representation for calculation of actor overlap Ω at the scale $a = 8$ days, $b \approx 1408$ km. At the given scale we observe only three conflict zones in Africa.

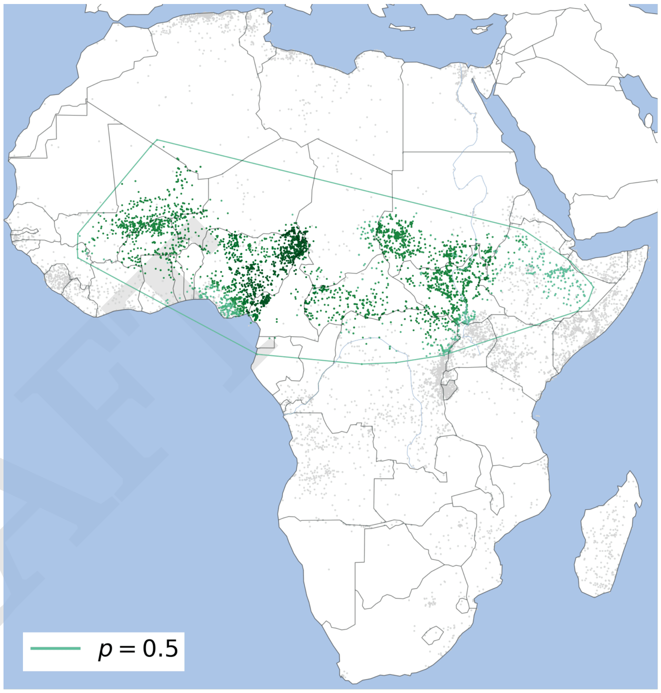

Fig. S10. Conflict interaction zone for temporal scale $a = 64$ days, spatial scale $b \approx 352$ km, which is at the very top of the mesoscale in Figure 3.

are equal to one. The actor overlap score Ω is calculated by taking the mean over all the elements of the matrix M (see Figure S9).

H. Statistical causality and transfer entropy

In 1956, Wiener formulated causality in terms of predictability: "For two simultaneously measured signals, if we can predict the first signal better by using the past information from the second one than by using the information without it, then we call the second signal causal to the first one" (9). Later, Granger formulated it mathematically by introducing a statistical concept of causality based on evaluation of predictability which is now commonly known as "Granger causality" (10). Transfer entropy is an information theoretic measure that generalizes some of the assumptions for Granger causality (GC). Namely, transfer entropy neither requires an explicit model (GC assumes a linear relationship between the predicted and predicting variables) nor assumes normality in their distributions.

In short, transfer entropy is an information-theoretic quan-

DRAFT

tity (typically based on Shannon entropy), which measures the flow of information between two or more time series which in turn is a measure for statistical causality. Recently, transfer entropy has been used widely as a measure of statistical causality while dealing with complex systems in the form of time series (11–13).

The transfer entropy considers the ratio of the conditional distribution of one variable x_{t+1} at a future time $t+1$ given its own past x_t and the past of a candidate causal process y_t versus the conditional distribution of that variable depending only on its own past values (14),

$$T[X;Y] = \sum_{x_t, x_{t+1}, y_t} q(x_t, x_{t+1}, y_t) \log\left(\frac{q(x_{t+1}|x_t, y_t)}{q(x_{t+1}|x_t)}\right). \quad [3]$$

Transfer entropy is an asymmetric quantity, i.e $T[X;Y] \neq T[Y;X]$, and therefore unlike time-delayed mutual information and correlation, transfer entropy can detect directional interactions which are a proxy for causal relationships. Importantly, the transfer entropy is by definition zero when two time-series are statistically independent, meaning that it can identify cases missing causal signatures.

I. Interpolating mesoscale

In our study, we analyze the range of spatial separation scales from $b \approx 22\,\mathrm{km}$ to $b \approx 1408\,\mathrm{km}$ and temporal separation scales from $a = 1\,\mathrm{day}$ to $a = 2^9$ days. Specifically, we calculate the spatial range at 13 marks separated by factors of $\sqrt{2}$. These are at about $b \approx 22, 33, 44, 66, 88, 132, 176, 264, 352, 528, 704, 1056, 1408$ km and the temporal range at 10 marks separated by factors of 2, $a = 1, 2, 4, 8, 16, 32, 64, 128, 256, 512$ days. Thus, we have 130 combinations of spatiotemporal scales. In Figure 3b and d, we perform calculations for 130 points in total and the rest of the space the values are computed using a bivariate spline approximation. Figure 3a is obtained by averaging the contours from Figure 3b and d for 100 realizations of the Voronoi tessellations. We obtain a smoothed boundary with a bicubic interpolation.

J. Scaling hypothesis

We check if the dynamical exponent relations relating each scaling variable with duration T as discussed in the main text are satisfied across 100 random realizations of the Voronoi tessellation. If the relation is satisfied for a given spatiotemporal scale within the mesoscale more than 90% of time, we conclude that the exponent relation is significant. The result of this analysis is shown in Figure S11.

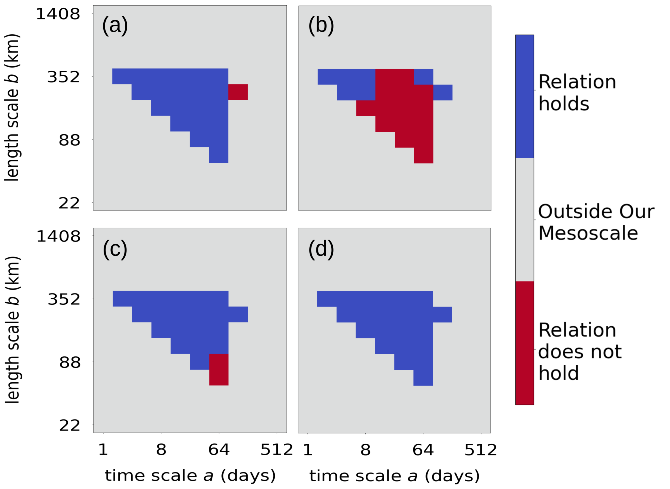

Fig. S11. Significance of dynamical exponent relations in the mesoscale for (a) fatalities, (b) reports, (c), sites, and (d) diameter. Each block corresponds to a spatiotemporal scale (see Appendix I for more details). Validity of exponent relation is checked for 100 pseudorandom Voronoi tessellation realization. If more than 95% of these random realizations satisfy the exponent relation, we conclude that the exponent relation is significant (blue); otherwise, it is not (red).

DRAFT

1. Raleigh C, Kishi R (2019) Similarities and Differences Across Conflict Datasets. p. 33.
2. Davies J (2014) Poisson-Disc Sampling (https://www.jasondavies.com/poisson-disc/).
3. Lee ED, Daniels BC, Myers CR, Krakauer DC, Flack JC (2020) Scaling theory of armed-conflict avalanches. *Phys. Rev. E* 102(4):042312.
4. Kushwaha N, Lee ED (2022) Visualization of ACLED dataset at fine grained level (temporal bin size=1 day) (https://www.youtube.com/watch?v=AJebsYGcQbU).
5. Kushwaha N, Lee ED (2022) Visualization of ACLED dataset at coarse-grained level (temporal bin size=64 days) (https://www.youtube.com/watch?v=drH1QOyIaM8).
6. Lee ED, Daniels BC, Myers CR, Krakauer DC, Flack JC (2020) Emergent regularities and scaling in armed conflict data. *arXiv:1903.07762 [cond-mat, physics:nlin, physics:physics, q-bio]*.
7. Clauset A, Shalizi CR, Newman MEJ (2009) Power-Law Distributions in Empirical Data. *SIAM Rev.* 51(4):661–703.
8. OECD, Club SaWA (2020) *Africa's Urbanisation Dynamics 2020.*
9. Wiener N (1956) *The Theory of Prediction. Modern Mathematics for Engineers.* (New York 165).
10. Granger CWJ (1969) Investigating Causal Relations by Econometric Models and Cross-spectral Methods. *Econometrica* Vol. 37(No. 3):424–438.
11. Vicente R, Wibral M, Lindner M, Pipa G (2011) Transfer entropy—a model-free measure of effective connectivity for the neurosciences. *J Comput Neurosci* 30(1):45–67.
12. Wibral M, et al. (2011) Transfer entropy in magnetoencephalographic data: Quantifying information flow in cortical and cerebellar networks. *Progress in Biophysics and Molecular Biology* 105(1-2):80–97.
13. Marschinski R, Kantz H (2002) Analysing the information flow between financial time series: An improved estimator for transfer entropy. *Eur. Phys. J. B* 30(2):275–281.
14. Schreiber T (2000) Measuring Information Transfer. *Phys. Rev. Lett.* 85(2):461–464.

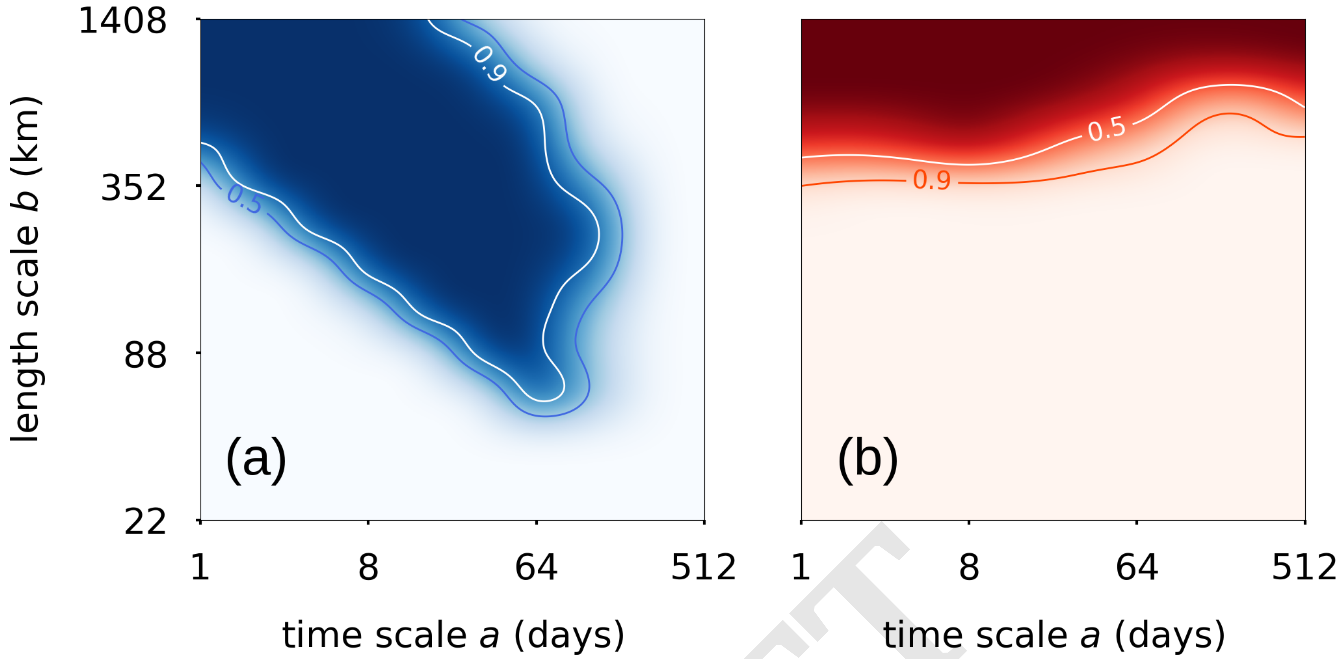

Fig. S12. Variability of mesoscale boundaries across 100 Voronoi tessellations to (a) percent data used Φ and (b) actor overlap Ω using as in the main text $\Phi \geq 3/4$ and actor overlap less than the midpoint $\Omega \lesssim 0.132 \pm 0.002$.

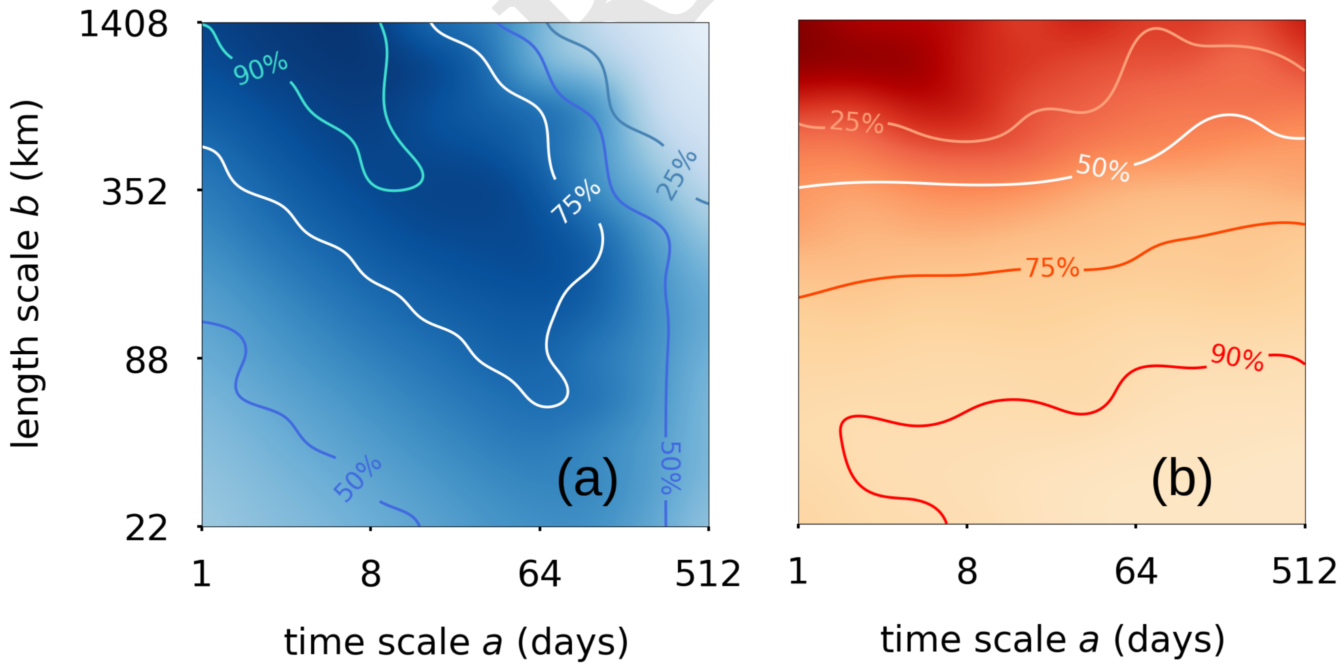

Fig. S13. Sensitivity of mesoscale boundaries to difference choices of (a) percent data used Φ and (b) actor overlap Ω. Each line indicates a different percentile threshold. White contour is the threshold reached from $a\ priori$ reasoning and used in the main text in Figure 3.

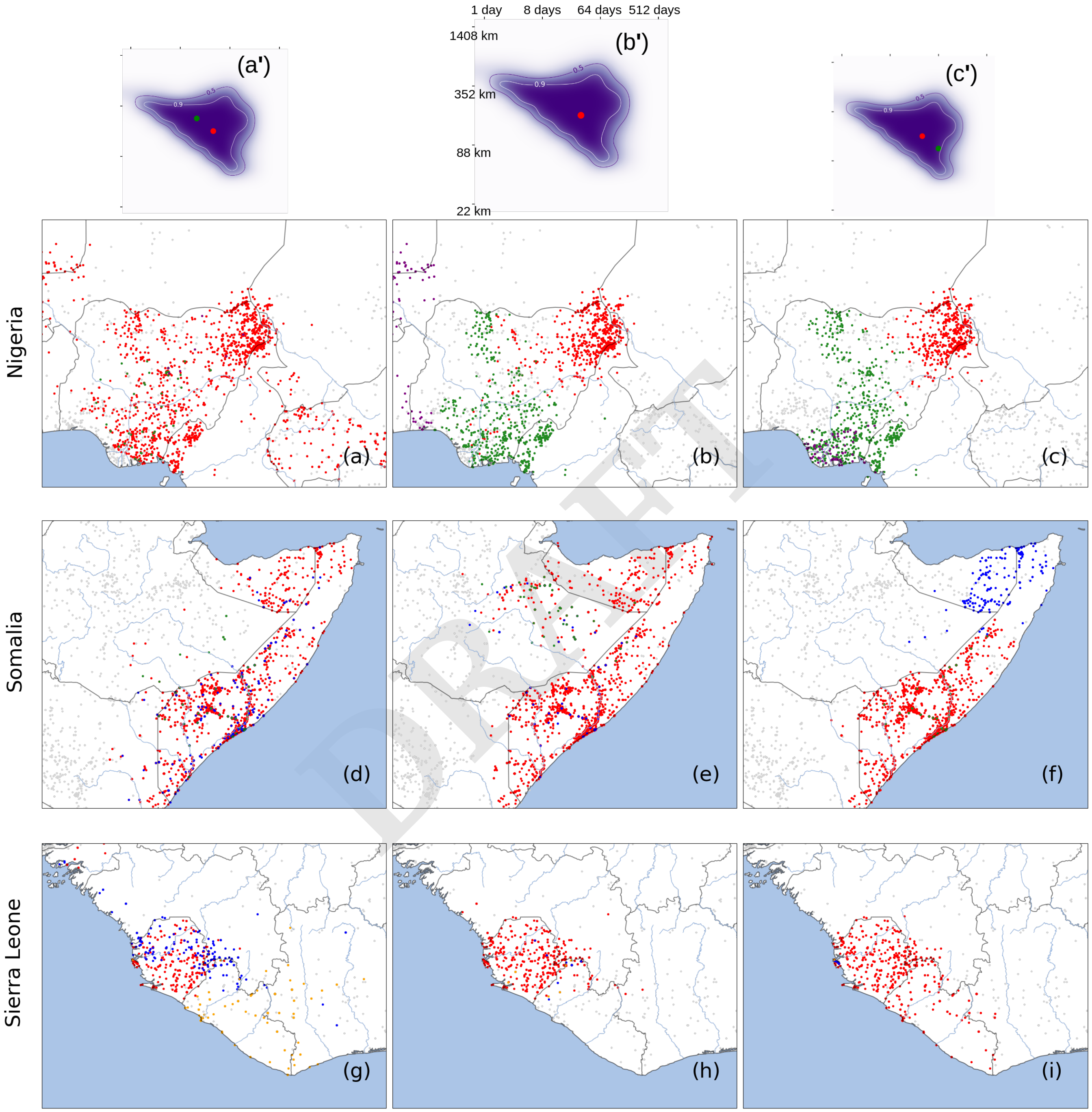

DRAFT

Fig. S14. Conflict avalanches from Figure 6 from a few different scales within the mesoscale. Each pane shows three biggest conflict avalanches (each with a different color) at the corresponding region and scale. Gray points are events not part of the highlighted avalanches. Conflict in (a,d,g) with $b \approx 246\,\text{km}$, $a = 16\,\text{days}$, (b,e,h) with $b \approx 180\,\text{km}$, $a = 32\,\text{days}$, and (c,f,i) with $b \approx 120\,\text{km}$, $a = 64\,\text{days}$. The mesoscales (a'), (b') and (c') show colored dots, depicting the scale at which corresponding avalanches (a,d,g), (b,e,h) and (c,f,i) are generated. The central red dot in (a') and (c') functions as a reference point for the green dots.

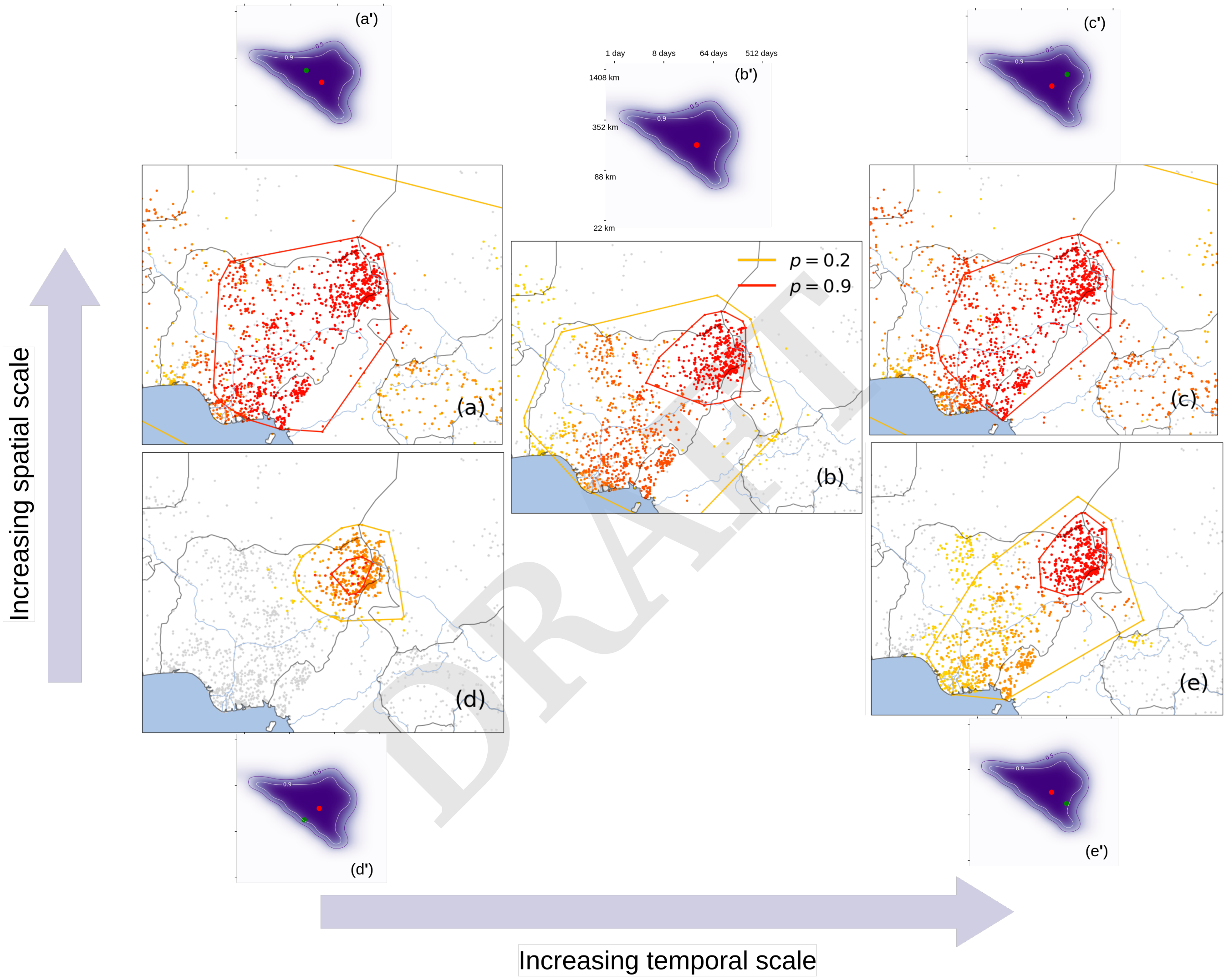

Fig. S15. Conflict interaction zones from Figure 6 for a few sample scales within the mesoscale. Nigeria with (a) $b \approx 246$ km, $a = 16$ days, (b) $b \approx 180$ km, $a = 32$ days, (c) $b \approx 246$ km, $a = 64$ days, (d) $b \approx 120$ km, $a = 16$ days, and (e) $b \approx 120$ km, $a = 64$ days. Probability of an event being part of the highlighted conflict avalanche is measured using 100 pseudorandom Voronoi tessellations. Gray points are events that are not part of the shown avalanche. Mesoscale legends (a'), (b'), (c'), (d') and (e') indicate with the green dot the corresponding scale. Central red dot serves as a reference point.

DRAFT
